# Free Cholesterol Bioavailability and Atherosclerosis

**DOI:** 10.1007/s11883-022-01011-z

**Published:** 2022-03-25

**Authors:** Rei J. Abe, Jun-ichi Abe, Minh T. H. Nguyen, Elizabeth A. Olmsted-Davis, Abrar Mamun, Priyanka Banerjee, John P. Cooke, Longhou Fang, Henry Pownall, Nhat-Tu Le

**Affiliations:** 1 Center for Cardiovascular Sciences, Houston Methodist Research Institute, Houston, TX USA; 2 Department of Physiology and Biophysics, Weill Cornell Medicine, New York, NY USA; 3 Department of Cardiology, The University of Texas MD Anderson Cancer Center, Houston, TX USA; 4 University of Science and Technology of Hanoi, Vietnam Academy of Science and Technology, Hanoi, Vietnam; 5 Weill Cornell Medicine, New York, NY USA; 6 Center for Bioenergetics, Department of Medicine, Houston Methodist Research Institute, Houston, TX USA

**Keywords:** Cardiovascular disease, Free cholesterol, High-density lipoprotein, Scavenger receptor class B type 1, Reverse cholesterol transport

## Abstract

**Purpose of Review:**

As both a cholesterol acceptor and carrier in the reverse cholesterol transport (RCT) pathway, high-density lipoprotein (HDL) is putatively atheroprotective. However, current pharmacological therapies to increase plasma HDL cholesterol (HDL-c) concentration have paradoxically failed to prevent or reduce atherosclerosis and cardiovascular disease (CVD). Given that free cholesterol (FC) transfer between surfaces of lipoproteins and cells is reversible, excess plasma FC can be transferred to the cells of peripheral tissue sites resulting in atherosclerosis. Here, we summarize potential mechanisms contributing to this paradox and highlight the role of excess free cholesterol (FC) bioavailability in atherosclerosis vs. atheroprotection.

**Recent Findings:**

Recent findings have established a complex relationship between HDL-c concentration and atherosclerosis. Systemic scavenger receptor class B type 1 (SR-B1) knock out (KO) mice exhibit with increased diet-induced atherosclerosis despite having an elevated plasma HDL-c concentration compared to wild type (WT) mice. The greater bioavailability of HDL-FC in SR-B1 vs. WT mice is associated with a higher FC content in multiple cell types and tissue sites. These results suggest that dysfunctional HDL with high FC bioavailability is atheroprone despite high HDL-c concentration.

**Summary:**

Past oversimplification of HDL-c involvement in cholesterol transport has led to the failures in HDL targeted therapy. Evidence suggests that FC-mediated functionality of HDL is of higher importance than its quantity; as a result, deciphering the regulatory mechanisms by which HDL-FC bioavailability can induce atherosclerosis can have far-reaching clinical implications.

## Introduction

While levels of cholesterol have been strongly associated with the risk of atherosclerosis and cardiovascular disease (CVD), it is essential to many biological functions as well, from cell structure and membrane fluidity to brain development. Cholesterol is a precursor in biosynthesis of bile, steroid hormones, vitamin D [1], hedgehog protein, myelin, and other substances [2, 3], and contributes to the formation of lipid rafts, synapses, and dendrites. Cholesterol primarily localizes to membranes, and is involved in the restriction of the passage of positive hydrogen and sodium ions across the cell membranes in nerve conduction [4–6], and facilitates proper cell signaling [7]. Through interaction with other lipids in the bilayer, cholesterol is also involved in regulation of the rigidity and permeability of cell membranes [8, 9]. As a result of its involvement in a diverse number of crucial physiological functions, defective cholesterol metabolism can result in an increased risk of a number of pathological disorders. These include atherosclerosis, CVD [10], and central nervous system diseases (Smith-Lemli-Opitz syndrome [11], Niemann-Pick type C disease [12], Huntington’s disease [13], and Alzheimer’s disease) [14].

## Cholesterol Metabolism

**Fig. 1:**
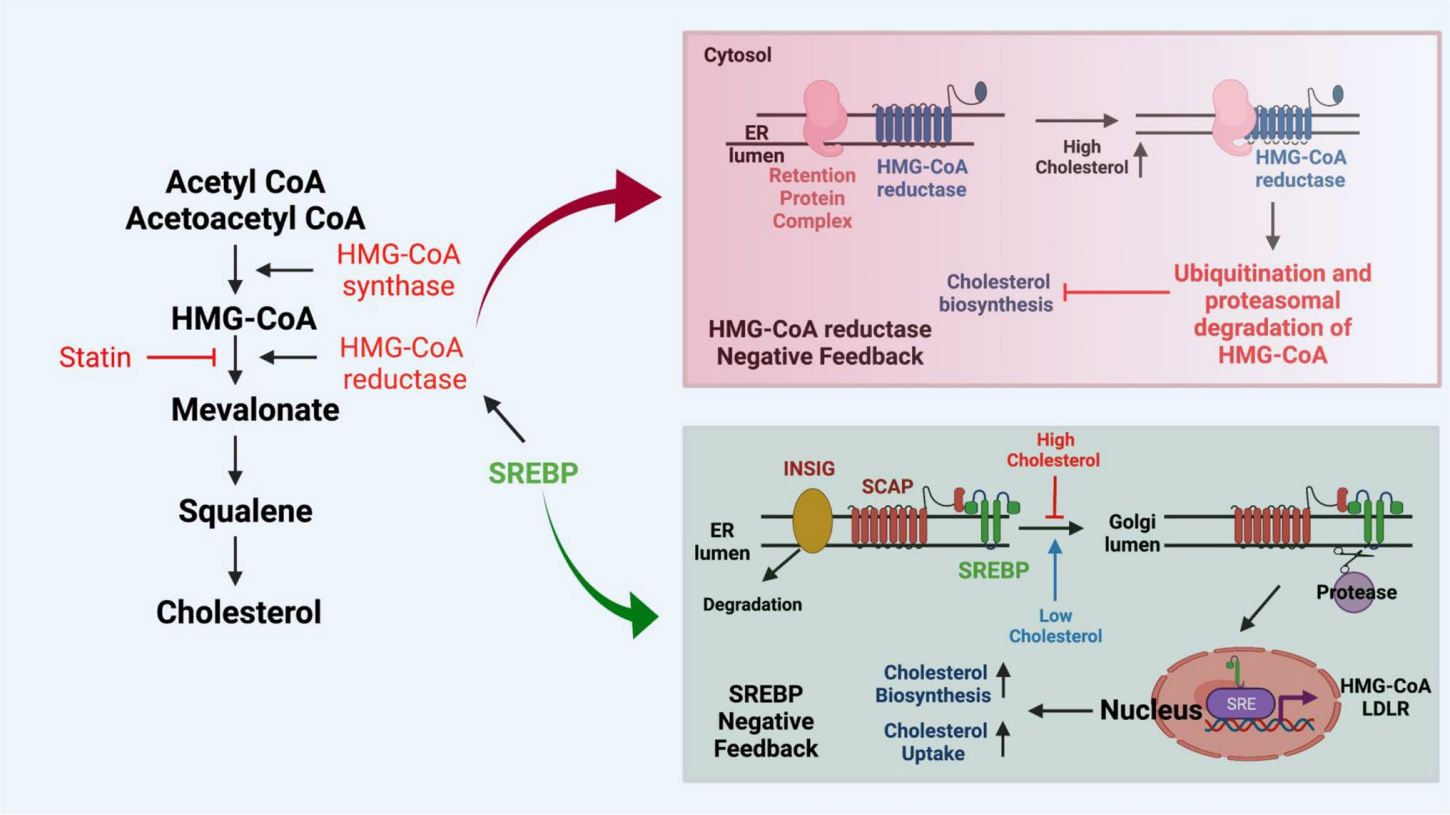
Cholesterol biosynthesis. **Initiation**: Acetyl-CoA and acetoacetyl-CoA are dehydrated by HMG-CoA synthase in order to form HMG-CoA, which is then reduced to form Mevalonate by HMG-CoA reductase. Statins, which are commonly used to lower cholesterol, inhibit HMG CoA reductase. Mevalonate is condensed to squalene, a biochemical precursor for cholesterol. **HMG-CoA reductase negative feedback regulation**: HMG-CoA reductase in the ER is regulated in a negative feedback loop by intracellular cholesterol content. As cholesterol content increases, HMG-CoA reductase undergoes membrane domain changes leading to its ubiquitination and proteasomal degradation, resulting in the inhibition of cholesterol biosynthesis. **SREBP negative feedback regulation**: When intracellular cholesterol is high, the SREBP-SCAP complex remains in the ER. However, when cholesterol levels decrease, SREBP-SCAP is then transported to the Golgi, where SREBP undergoes cleavage, allowing it to enter the nucleus and activate genes involved in cholesterol biosynthesis, increasing HMG-CoA, as well as cholesterol uptake via the LDL receptor. The subsequent induction of cholesterol biosynthesis results in an increase in intracellular cholesterol, and once again inhibits SREBP-SCAP transport to the Golgi, repressing SREBP activation

The current understanding of cholesterol metabolism begins with the metabolite acetyl coenzyme A (acetyl-CoA). Through enzymatic reactions mediated by the integral endoplasmic reticulum (ER) membrane protein 3-hydroxy-3-methylglutaryl CoA (HMG-CoA) reductase enzyme, as well as mevalonate kinase, cholesterol is synthesized in the ER [10]. Acetyl CoA and acetoacetyl-CoA are dehydrated to form HMG-CoA, which is reduced to mevalonate by HMG-CoA reductase enzyme, a step that is blocked by HMG CoA reductase inhibitor, or statins (Fig. 1). Cholesterol biosynthesis is controlled by a negative feedback mechanism involving sterol regulatory element-binding proteins 1 and 2 (SREBP1, 2). Low intracellular cholesterol concentrations increase cholesterol biosynthesis, which is then suppressed by elevated cellular cholesterol concentrations. Mechanistically, elevated cholesterol concentrations induce changes in the HMG CoA reductase membrane domain that make it susceptible to degradation (Fig. 1). Upon degradation of HMG CoA reductase, cholesterol biosynthesis is inhibited, resulting in decreased levels of cholesterol, which then promotes HMG CoA reductase production. Similarly, when intracellular cholesterol levels are high, SREBP is localized in the ER membrane and bound to sterol regulatory element binding protein cleavage activating protein (SCAP). At these cholesterol levels, SCAP undergoes a conformational change and binds to insulin-induced gene proteins (INSIG), keeping SREBP-SCAP in the ER. However, in low cholesterol levels, INSIG-SCAP binding is disrupted, allowing for SREBP to be transported from the ER to the Golgi apparatus by SCAP [15–17]. In the Golgi, SREBP is cleaved, processed, then sent to the nucleus where it activates genes related to cholesterol biosynthesis, leading to anincrease in HMG CoA, as well as cholesterol uptake through the low-density lipoprotein receptor (LDLR). The resulting increase in cholesterol then once again inhibits Golgi transport of SREBP-SCAP, resulting in inhibition of SREBP activation (Fig. 1). A genetic defect of cholesterol biosynthesis linked to mevalonate kinase deficiency, identified in 1986, is the only recognized disorder that belongs to the first half or ‘pre-squalene’ portion of human cholesterol biosynthesis, although a targeted mutation in mouse squalene synthase gene is associated with embryonic lethality and defects of neural tube closure [18–22]. AMP-activated protein kinase can phosphorylate and inhibit HMG CoA reductase activity [23]. Whole-body cholesterol is obtained endogenously and from dietary intake. The liver is the primary endogenous source and synthesizes 20 ~ 25% of total cholesterol daily; the adrenal glands, intestines, and reproductive organs synthesize smaller amounts. Since tissues can synthesize enough cholesterol for their various biological functions, cholesterol from food ingestion is not required for maintenance of homeostasis.

In cholesterol metabolism, dietary cholesterol, lipids, and triglycerides (TG) in the intestine are assembled into chylomicrons that are transported to the liver. Free fatty acids released from chylomicron-TG particles are taken up by muscle and adipose tissues. In the liver, cholesterol, endogenous- and dietary-TG, and other lipids are assembled into apolipoprotein-B (APOB)-containing lipoproteins and transported to peripheral tissues through the circulation. As the lipid-to-protein ratio increases, the lipoprotein particle sizes and densities, respectively, increase and decrease. Lipoproteins are classified based on their density, with sizes consisting of very low-density lipoprotein (VLDL), intermediate density lipoprotein (IDL), low-density lipoprotein (LDL), and high-density lipoprotein (HDL). Each lipoprotein type contains different surface-proteins, which determine their specific functions. LDL particles have the highest cholesterol content, defined as the sum of free cholesterol (FC) and cholesteryl ester (CE), and are the major lipoprotein for the transport of cholesterol from the liver to peripheral tissues. VLDL-TG lipolysis by lipoprotein lipase (LPL) converts VLDL to IDL. IDL-TG undergoes lipolysis by hepatic lipase (HL), which converts IDL to LDL. Both these processes release free fatty acids into the circulation [24, 25]. A CE transfer protein (CETP) exchanges HDL-CE for the TG of APOB-containing lipoproteins [26]. These lipoproteins, if chemically modified, are taken up by macrophages, forming foam cells [27]. Excess plasma LDL transferred to the subendothelial space of the arterial wall is atherogenic but can be reduced by LDL-lowering therapies, especially statins. HDL particles vary in sizes, densities, and lipid-protein composition [28]. Spherical α-HDL particles are CE-rich, larger, but less dense vs. pre-β-HDL, which are lipid-poor, smaller, and denser [29, 30]. Results from Gofman, Miller, and Miller, as well as the Framingham Heart Study, revealed an inverse correlation between plasma HDL-cholesterol concentrations and CVD risk [31–33]. From this seminal discovery, HDL was identified as a key player in the reverse cholesterol transport (RCT), i.e., the transfer of peripheral tissue cholesterol, including the arterial wall, to the liver for disposal. As both a cholesterol acceptor and carrier, HDL facilitates cholesterol efflux from peripheral, extra-hepatic, and arterial tissues, including cholesterol-loaded monocyte-derived macrophages and foam cells, back to the liver to protect the cells from toxicity induced by FC and oxysterol. In the liver, FC is excreted either as biliary cholesterol or converted to bile acids or recycled.

### The Biliary Pathway of FC Efflux

**Fig. 2:**
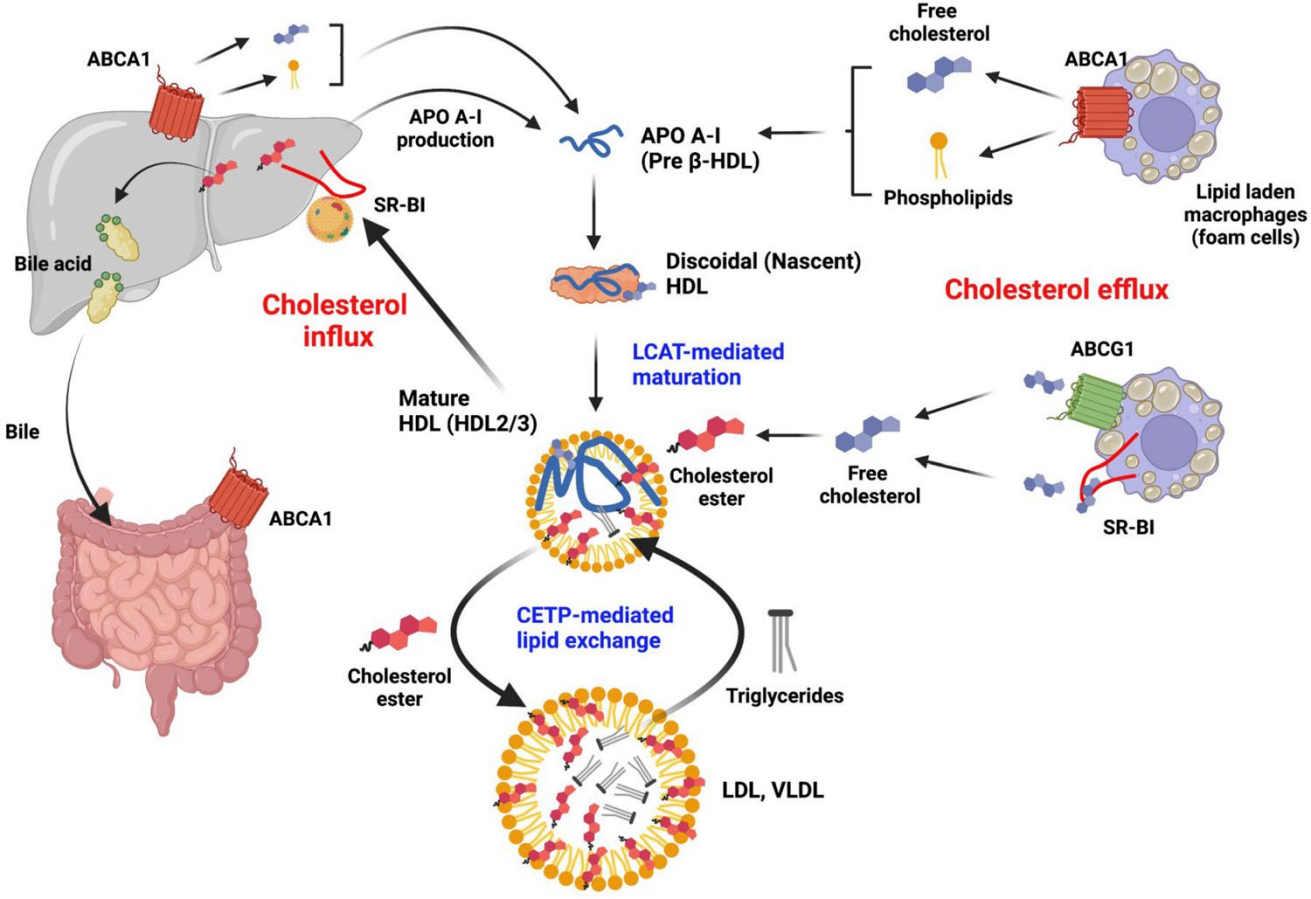
HDL-mediated reverse cholesterol transport. **Cholesterol efflux**: Macrophages localize to fat deposits on the walls of blood vessels, forming lipid laden foam cells — both foam cell and hepatic ABCA1 contribute to HDL formation. ABCA1 and APOA1 interaction induces transfer of FC and phospholipids to APOA1, forming nascent HDL. **LCAT mediated maturation**: LCAT converts HDLFC to CE and allows for more FC uptake on the HDL surface; FC is also transferred to HDL via ABCG1. LCAT mediated conversion prevents reverse uptake of FC by the macrophages from mature HDL, which then enters either the direct (SR-B1) or indirect (CETP) route. **Cholesterol influx**: Hepatic SR-B1 selectively extracts FC, CE, and phospholipids from mature HDL, generating APOA-1 which can re-enter the cycle. CE is hydrolyzed into FC and incorporated into bile acids which are secreted into the intestines. **CETP-mediated lipid exchange**: CETP transfers HDL-CE to VLDL and LDL in exchange for triglycerides. CE and FC associated with VLDL/LDL are then delivered to the liver by the LDL receptor. Created with BioRender.com

FC efflux involves macrophage-ATP binding cassette transporter A1 (ABCA1) and the major HDL-apolipoprotein, APOA1 (Fig. 2). The ABCA1-APOA1 interaction mediates cholesterol and phospholipid transfer to APOA1, thereby forming nascent HDL particles (called pre-β HDL) that contain FC, phospholipid, and APOA1. Subsequently, pre-β HDL binds the sub-family G member 1 (ABCG1), which increases pre-β HDL FC content, forming mature HDL (α-HDL)[27]. Lecithin-cholesterol acyltransferase (LCAT) converts α-HDL-FC to CE and frees spaces on the HDL surface for FC uptake. The RCT pathway involves direct and indirect routes [34–37]. In the direct route, cholesterol influx to the liver is mediated by the HDL receptor scavenger receptor Class B Type 1 (SR-B1) (Fig. 2). The liver then extracts α-HDL-CE, FC, and phospholipids, thereby generating lipid-poor APOA1 and cholesterol-depleted HDL, both of which can re-enter the HDL/APOA1 cycle [26]. Following SR-B1-induced cholesterol uptake, CE is hydrolyzed to FC for bile acid production, which is either secreted into the intestine (to break down fats) excreted with the feces, which reduces whole-body FC), or recycled to the liver. SR-B1I, an SR-B1 alternative splicing isoform with the C-terminus cytoplasmic domain almost completely altered, is also expressed in caveolae and reduces plasma HDL cholesterol. SR-B1I mediates both selective cellular CE uptake and HDL-dependent cholesterol efflux from cells with around fourfold less efficiency as compared to SR-B1[38]. In the indirect route, CETP-mediated lipid exchange transfers HDL-CE to VLDL [26] in exchange for TG, and the liver extracts cholesterol (CE and FC) from these particles via the LDL receptor (LDLR), which binds its APOB100 component (Fig. 2). Unlike the SR-B1-dependent mechanism, LDLR-mediated cholesterol uptake leads to lysosomal-induced degradation of lipoproteins to release cholesterol. This indirect route transfers ~ 70% of total CE back to the liver. Additionally, hepatic LDLR is also responsible for the direct uptake of APOE-containing HDL particles, which occur as both HDL_2_ and HDL_3_ and may be derived either by transfer from TG-rich lipoproteins or from tissue sources (liver and monocyte-macrophages). In addition to the two RCT routes, CE can be delivered back to the liver via the holo-particle uptake; however, the exact molecular mechanism in this transfer has not yet been identified.

### The Non-biliary Pathway of FC Efflux

HDL/SR-B1 interaction and the subsequent selective CE uptake are central to the HDL-mediated hepato-biliary RCT pathway [39–49]. According to this widely accepted mechanism, elevated plasma HDL concentrations might be expected to irreversibly remove excess cholesterol and thereby prevent cholesterol influx into the arterial wall. Accordingly, increasing HDL concentrations seemed a promising antiatherogenic approach. However, currently, no studies using HDL therapy have been atheroprotective. Pharmacological induction of plasma HDL failed to prevent or reduce CVD [50–52]. Clinical trials failed to capture the efficacy of HDL-raising therapies on CVD [50, 53]. Human genetic studies suggested that plasma HDL concentrations do not necessarily reflect its overall abundance, subspecies distribution, cholesterol efflux capacity, or are predictive of CVD risk. As such, further studies are required to completely understand the regulator mechanisms controlling cholesterol efflux. Besides the HDL-mediated hepato-biliary RCT pathway [54–59], cholesterol efflux can occur via a non-biliary pathway, or the non-hepatic transintestinal cholesterol excretion (TICE) pathway [60]. TICE involves the direct secretion of plasma lipoprotein-cholesterol by the small intestine [61–64] via an unknown mechanism [44, 65]. Hepatic HDL binds SR-B1 and facilitates the HDL-mediated hepato-biliary RCT pathway, sending CE back to the liver for biliary disposal [41]. Hepatocyte-specific SR-B1 knock out (KO) mice exhibit more severe atherosclerosis and early death due to coronary artery occlusions and fibrotic myocardial lesions [66]. In mice, hepatic SR-B1 overexpression increases CE selective uptake from HDL-CE and macrophage-to-feces RCT [67, 68]. SR-B1 is also expressed in enterocytes and small intestine-specific SR-B1 overexpression in mice reduces plasma HDL-C concentrations but does not change cholesterol absorption [69]. Treatment of mice with small intestine-specific SR-B1 overexpression with ezetimibe, which blocks cholesterol absorption, did not affect fecal neutral sterol excretion, suggesting that SR-B1 overexpression in the intestine does not increase TICE. In their review, Temel et al. have proposed a new integrated model of the non-biliary TICE and RCT pathway as well as strategies to stimulate intestinal cholesterol excretion for the prevention or treatment of CVD [60].

## SR-B1 Is One of the Key Components in the Biliary Pathway of FC Efflux

SR-B1 is a member of the scavenger receptor class B family, which also includes CD36 and CD36 antigen-like II (LIMPII)[70]. This 82-kDa membrane glycoprotein was identified by its homology to CD36. Like LIMPII and CD36, SR-B1 has 2 transmembrane domains linked to 2 intracellular N- and C-termini and an extracellular glycosylated central domain separated by a large extracellular loop. SR-B1 binds various ligands including acetylated, oxidized, and native VLDL, LDL, HDL [71], as well as Lp(a), modified serum proteins such as maleylated bovine serum albumin, advanced glycation end-product modified proteins, anionic phospholipids, lipid vesicles containing negatively charged phospholipids, and apoptotic cells [71–74]. SR-B1 binds apolipoproteins on the HDL surface, including APOA1[75–79]. This association of SR-B1/APOA1 requires specific structural domains (the amino- or the carboxy-terminal amphipathic helices) [30] and conformation states of APOA1 on HDL particles [80]. Relative content of APOA1 and -2 in spherical HDL particles also affects the SR-B1/APOA1 association [81–84]. Moreover, the conformation and organization of APOA1 on HDL particles is crucial to SR-B1 binding to HDL, facilitating the transport of lipids (CE, phospholipids, TG, and lipid soluble vitamins). Binding of SR-B1 to the reconstituted discoidal APOA1 complexes is stronger than to α-HDL, whereas its binding to α-HDL is stronger than to pre-β-HDL [29, 30]. While forming complexes with these lipoproteins/receptors including mature α-HDL particles, SR-B1 facilitates cholesterol transfer between HDL-cholesterol (HDL-C) and cell membranes, mediates a selective CE uptake from HDL-CE without particle uptake, and promotes the elimination of excess cholesterol via biliary cholesterol secretion. Unlike ABCA1 and ABCG1, which mediates cholesterol transport in a passive, concentration-dependent, and energy-independent manner, SR-B1 mediates bidirectional cholesterol flux.

## SR-B1 in Atherosclerosis.

### Hepatic and Extra-hepatic SR-B1 Is Atheroprotective

SR-B1 binds a multi-PDZ domain containing adaptor protein PDZK1, which can regulate SR-B1 protein expression levels [85]. In PDZK1 KO mice, the SR-B1/PDZK1 complex controls SR-B1 expression in a tissue-specific and post-transcriptional dependent manner. SR-B1 expression is reduced by 95% in the liver and 50% in the proximal intestine of the PDZK1 KO, as compared to that of the WT mice. However, SR-B1 expression level is unaltered in steroidogenic organs (adrenal, ovary, and testis). In hepatocytes, expression of a SR-B1 splice variant with an alternative C-terminal cytoplasmic domain (SR-B1I) is not affected in PDZK1-KO mice, suggesting that the PDZK1/SR-B1 complex is required for hepatic SR-B1 expression [86].

Plasma cholesterol concentrations were increased in SR-B1 targeted heterozygous (HET, 31%) and homozygous KO (KO, 125%) mice due to the formation of large particles containing APOA1, while alternatively, cholesterol content in adrenal glands were decreased in HET (42%) and KO (72%) [87]. Since plasma APOA1 concentration was unaltered, the data suggests that the increased plasma cholesterol is due to defective HDL-C uptake [87]. Among SR-B1 KO mice, SR-B1 deficiency [87] differentially alters lipid metabolism in arterial and hepatic cells [88]. SR-B1 KO vs. WT mice fed a regular diet (4.3% fat, no cholesterol) exhibit a 1.8-fold increase in total serum cholesterol (TC) due to a 3.2-fold increase in HDL-FC [88]. Changes in TC concentrations in SR-B1 KO mice are due to an increased HDL-C. No significant difference in cholesterol profile between SR-B1 HET and WT was noted, except for a slightly increased VLDL-C concentration. After 20 weeks of feeding a high cholesterol diet (HCD, 15% fat, 0.25% cholesterol), plasma HDL-C of SR-B1 KO mice was greatly increased with minor changes in VLDL-C concentrations, and compared to the WT mice, the KO mice developed more severe atherosclerosis [88]. HDL compositions were also different in SR-B1 KO vs. WT, 9.6% and 4.5% for FC, 32.7% and 18.8% for CE, and 23.6 and 40.6% for phospholipid respectively. The protein contents of HDL from WT and SR-B1 KO mice were similar. Collectively, the data suggests that a HCD increases TC, HDL-FC, and HDL-CE and reduces phospholipids in SR-B1 KO mice without changing the serum TG concentrations and protein content [88]. Four hours after an initial injection of ^3^H-labeled HDL, 3.3 ± 0.8% of the injected dose was recovered in the liver of SR-B1 KO as compared to 31.3 ± 4.5% for WT mice. After correction for the HDL-CE pool size, the HDL-CE mass influx into the liver was 4.1 ± 0.7 μg/h in KO compared to 22.0 ± 3.8 μg/h in WT mice. This data suggested that the RCT pathway is deficient in SR-B1 KO mice, leading to the accumulation of HDL-FC [88]. This data also revealed the role of hepatic SR-B1 in the overall HDL-C metabolism and atherosclerosis [88]. The role of hepatic SR-B1 in whole particle uptake was confirmed using ^125^I-labeled tyramine cellobiose-labeled HDL. In SR-B1 KO mice, neither the hepatic cholesterol content (hepatic concentrations of FC, CE, and TG) nor the expression of key regulators of hepatic cholesterol homeostasis (HMG-CoA reductase, LDLR, and cholesterol 7α-hydroxylase) was altered, but biliary cholesterol content decreased ~ 40% [87, 89]. Conversely, expression of genes mediating inflammation and the dysregulation of cholesterol homeostasis in the arterial wall were increased, resulting in an enhanced lipid deposition in their aortas.

Mechanistically, hepatic SR-B1 mediates selective hepatic uptake of CE in HDL-CE via the RCT pathway, thereby trafficking CE to bile for excretion, while mediating cholesterol transfer for steroid hormone synthesis in the adrenal glands, ovaries, and testes [71, 79]. RCT is comprised of two steps (i) formation of an HDL/SR-B1 complex in an APOA1-dependent manner, and (ii) the transfer of CE from HDL-CE to the plasma membranes, internalized via a non-endosomal pathway and hydrolyzed by neutral CE via mechanisms yet to be characterized. CE hydrolysis produces FC that is used to produce bile, which is partly secreted into the intestine (to break down fats) and to the feces for excretion or recycled to the liver. Bile acid biosynthesis, the main pathway of cholesterol catabolism, is tightly regulated by the expression of the liver-specific enzyme cholesterol-7- α-hydroxylase (CYP7A1)[90]. In some species, excess cholesterol consumption leads to enhanced CYP7A1 expression and increased bile acid biosynthesis. Conversely, mice and hamsters that lack liver-X-receptor-α are unable to induce CYP7A1 in response to dietary cholesterol, and therefore are susceptible to cholesterol accumulation in plasma lipoproteins and liver, suggesting that modulation of the cholesterol-induced bile acid biosynthesis is important for the maintenance of cholesterol homeostasis. Reduced bile acid synthesis or dysfunctional biliary cholesterol secretion is associated with cholelithiasis and atherosclerosis [90]. The major cholesterol transport proteins including ABC transporters mediating biliary secretion of bile acids (ABCB11), phospholipids (ABCB4), and cholesterol (ABCG5/G8), and others including SR-B1, the phosphatidylserine flippase ATP8B1, and NPC1L1, modulate biliary cholesterol secretion [91]. Among SR-B1 KO mice, there was reduced expression of ABCG5 (70%) and ABCG8 (35%), suggesting that ABCG5/G8 is involved in SR-B1-mediated HDL-FC uptake and biliary cholesterol secretion.

The conditional SR-B1 KO mice, achieved by flanking exon 1 with Cre recombinase *lox*P sites to produce a hypomorphic allele (hypomSR-B1 KO)[92], exhibit twofold increase in plasma TC concentrations and 2.5-fold increase in atherosclerosis, as compared to the WT [92]. Hepatocyte-specific SR-B1 KO mice induced a hypomorphic SR-B1 allele inactivation in hepatocytes (hypomSR-B1–KO^liver^) and showed higher plasma TC concentration and FC/TC ratio as compared to the WT. The lipoprotein-cholesterol profile in these mice was similar to those of SR-B1 KO mice [87]. Plasma TC concentrations were increased twofold in hypomSR-B1 and control mice fed a HCD, while hypomSR-B1–KO^liver^ and SR-B1 KO mice developed severe hypercholesterolemia due to accumulation of FC-rich, VLDL-sized particles. Atherosclerosis in hypomSR-B1 mice was increased 2.5-fold compared to the control but was reduced 32- and 48-fold compared to hypomSR-B1–KO^liver^ and SR-B1 KO, respectively [87]. Plasma lipid concentrations or the capacity of VLDL-sized lipoproteins to induce macrophage cholesterol loading were similar for hypomSR-B1–KO^liver^ and SR-B1 KO mice, although atherosclerosis was reduced in hypomSR-B1–KO^liver^ due to a decrease in macrophage content compared to SR-B1. These results suggest that SR-B1 in extra-hepatic tissues is also atheroprotective [92, 93]. In ApoE KO mice, SR-B1 deletion (SR-B1/ApoE dKO) produces severe hypercholesterolemia (≈1000 mg/dl) with a significant increase in lipoprotein-FC and HDL particles in the size range of LDL and VLDL [94, 95], with defects in morphology and function like those seen in human congenital heart disease. When fed a regular diet, SR-B1/ApoE dKO mice display atherosclerosis in the aortic sinus and the mice possess lipid-rich coronary artery occlusions, myocardial fibrosis, and cardiac dysfunction (enlarged hearts, reduced ejection fraction and contractility, and abnormal echocardiography) at 4 ~ 5 weeks after birth. Accelerated atherosclerosis in SR-B1/ApoE dKO mice, partly due to abnormal lipoproteins [96, 97], leads to coronary artery occlusion, MI, and premature death 5 ~ 8 weeks after birth [66, 88, 94]. Coronary arterial lesions in SR-B1/ApoE dKO mice show evidence of cholesterol clefts and fibrin deposition, suggesting hemorrhage and clotting as seen in human atherosclerotic plaques. By 6 and 8 weeks of age, mortality was 50% and 100%, respectively [66]; however, longer-term high fat diet is required to induce coronary atherosclerosis in the SR-B1/ApoE-dKO mice. To induce myocardial infarction (MI), additional hypoxic or mental stress is needed and only occurs in 40% of the SR-B1/ApoE dKO mice [66, 93]. Similarly, Fuller et al. [98] showed that compared to LDLR KO, SR-B1/LDLR dKO mice exhibit abnormal lipoprotein profiles, severe coronary atherosclerosis, MI, and decreased survival rate (average lifespan = 9.4 weeks) when fed a modified Western diet containing high cholesterol (1.25%). Altogether, these data indicated that SR-B1 in hepatic and extra-hepatic tissues is atheroprotecive.

### Macrophage SR-B1 Is Atheroprotective

In humans, SR-B1 is abundantly expressed in tissues involved in a selective CE uptake with strong effects on HDL metabolism (adrenal glands, liver, steroidogenic tissues, etc.). SR-B1 is expressed in various cell types including hepatocytes, macrophages, endothelial cells, and enterocytes. Single-nucleotide polymorphisms (SNPs) in conserved regions of human SR-B1 extracellular domain are associated with impaired macrophage cholesterol efflux and platelet function, and increased CVD risk [99–103]. In mice on both LDLR KO and APOE KO background, the depletion of macrophage SR-B1 promotes atherosclerosis via a mechanism independent of its function as a hepatic HDL receptor [104, 105]. In atherosclerotic lesions, macrophages of SR-B1 KO mice are proinflammatory and susceptible to apoptosis [106]. SR-B1 in macrophages localizes to autophagosomes, forming cholesterol domains, increasing the recruitment of Barkor and the VPS34/Beclin-1 complex. By activating PPARα, macrophage SR-B1 upregulates autophagy regulator transcription factor EB (TFEB) and the VPS34/LC3/ATGs expression and thereby activating autophagy. Accordingly, SR-B1 depletion leads to the defective autophagy by inhibiting VPS34 activity in macrophages, increasing foam cell formation (1.8-fold), apoptosis (2.5-fold), and oxidized LDL-induced inflammatory cytokine expression, whereas TFEB or VPS34 overexpression reverses these processes [107].

### Endothelial SR-B1 Is Contextually Atheroprone

**Fig. 3:**
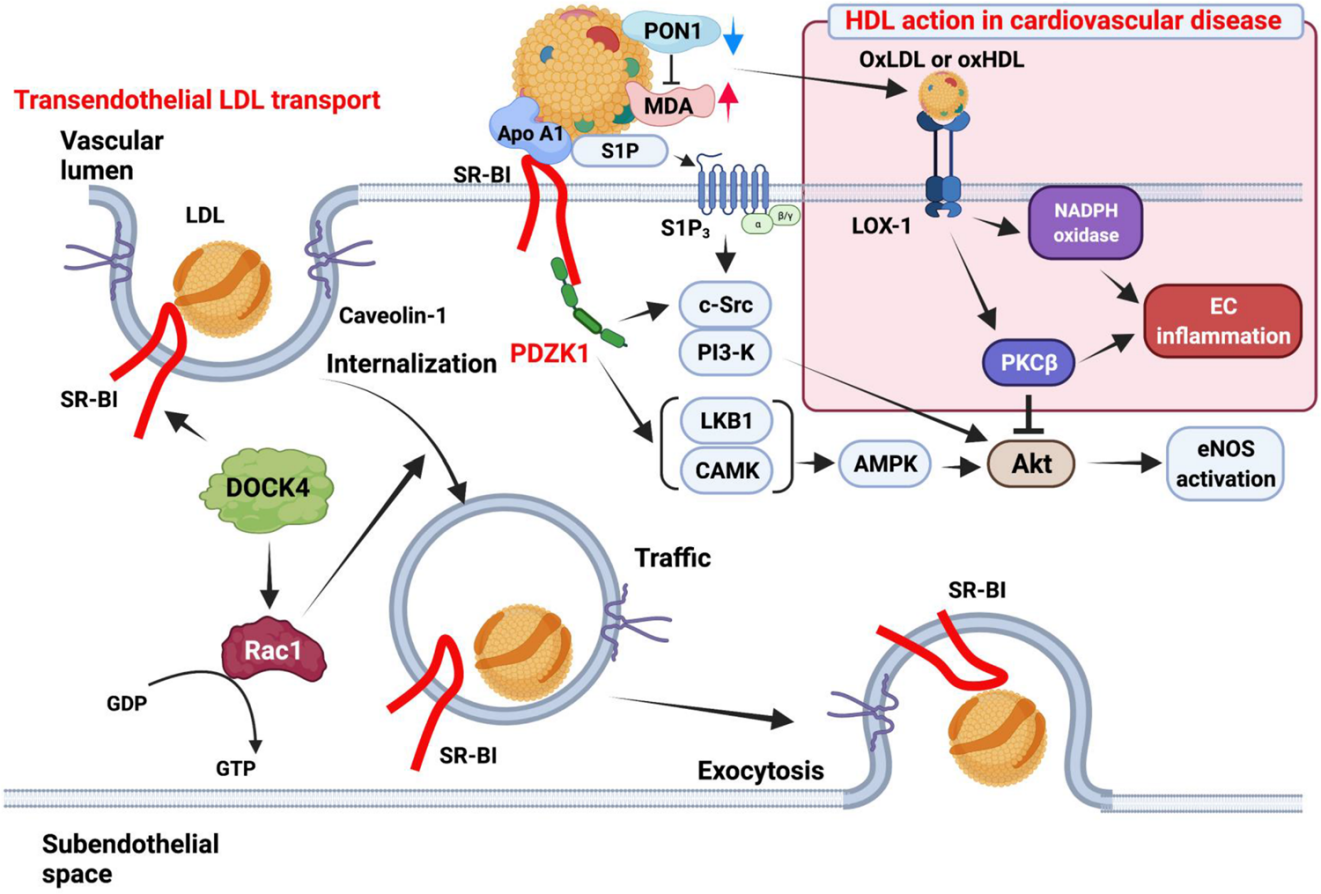
Overview of SR-B1-dependent signaling and mechanisms in endothelial cells. **Healthy HDL action in endothelial cells**: HDL can stimulate eNOS activation through several mechanisms. HDL binding to SR-B1 induces efflux of cholesterol and leads to adaptor protein PDZK1 interaction-dependent signaling; activation of non-receptor tyrosine kinase c-SRC leads to activation of PI3K, resulting in activation of Akt and eNOS. S1P sequestered in HDL can interact with S1P3 receptors, also resulting in P13K/Akt/eNOS activation. SR-B1/PDZK1 signaling also results in LKB1 and CAMK mediated activation of AMPK, inducing Akt and eNOS activation. HDL particles carry active PON1 enzyme which hydrolyze biologically active lipid peroxidases, thus suppressing oxidation of lipids and lipoproteins. This results in diminished levels of MDA and oxidative stress. **Transendothelial LDL transport**: In ECs, guanine nucleotide-exchange factor (GEF) DOCK4 couples with SR-B1 and is required for transcytosis of LDL. LDL binding with SR-B1 leads to interaction between SR-B1 and DOCK4, as well as activation of its associated Rho GTPase Rac1, resulting in caveolin-1 mediated transcytosis. Caveolae formation internalizes SR-B1 along with LDL, leading to trafficking of LDL to the subendothelial space where it contributes to foam cell and atherosclerotic plaque formation. **HDL action in cardiovascular disease**: HDL inhibits eNOS activation and induces ROS formation in cardiovascular disease. HDL in CVD has significantly reduced levels of PON1 activity, resulting in high levels of MDA and oxidation of lipoproteins and lipids. Oxidized lipoprotein can then activate LOX-1 which in turn stimulates PKCβ and inhibits Akt signaling. eNOS activity is reduced as a result. LOX-1 activation also regulates NADPH oxidase activity, inducing ROS generation. Decreased generation of the anti-atherosclerotic NO by eNOS and increased ROS generation results in promotion of EC inflammation and atherogenesis. Created with BioRender.com

Abundantly expressed in endothelial cells (ECs) [108–110], EC SR-B1 bound HDL is involved in HDL-mediated protective effects, such as increasing endothelial NO synthase (eNOS), EC repair, and anti-inflammatory processes [66, 111]. Healthy HDL function in endothelial cells is able to induce eNOS through several mechanisms. PDZ domain conatining 1 (PDZK1) interaction-dependent signaling results in proto-oncogene tyrosine-protein kinase Src (c-Src)-dependent activation of phosphoinositide 3-kinases (PI3K), leading to protein kinase B (Akt) and eNOS. Similarly, sphingosine 1-phosphate (S1P) in HDL can interact with sphingosine-1-phosphate receptor 3 (S1P3R), leading to P13K/Akt/eNOS activation. Lastly, paraxonase 1(PON1) enzyme in HDL particles are able to suppress oxidation of lipids and lipoproteins, diminishing malondialdehyde (MDA) and oxidative stress [112–114] (Fig. 3).

However, despite this seemingly atheroprotective function, EC-specific SR-B1 KO mice generated on an ApoE KO background showed a marked reduction of atherosclerosis in both males and females in both mixed and C57BL/6 background, while plasma TC, TG, HDL, and lipoprotein profiles were unaltered [66]. Transgenic mice expressing endothelial SR-B1, both C57Bl/6 N and ApoE-KO backgrounds, possess a slight decrease in plasma lipoprotein profiles [108]. TG and HDL-C concentrations are 1.2- and 1.35-fold lower, respectively, in the EC-specific SR-B1 deficient mice (C57Bl/6 N background) as compared to sibling controls. However, no differences were noted in plasma cholesterol between the EC-specific SR-B1 deficient mice and female non-transgenic siblings [108]. Endothelial SR-B1 expression had little effect on plasma lipoprotein concentrations in mice fed a regular diet, but it protected mice from diet-induced atherosclerosis by reducing plasma cholesterol, while increasing plasma HDL-C concentrations. In 8-month-old APOE-KO mice fed a regular diet, EC-specific SR-B1 expression decreases aortic lesions by 24%. Mice expressing SR-B1 in ECs and in the liver had a 1.5 ± 0.1-fold increase in plasma cholesterol compared to mice expressing SR-B1 only in the liver. This elevation was mostly due to increased HDL-C. In EC culture studies, SR-B1 was present in both basolateral and apical membranes but greater cellular uptake of cholesterol from HDL was found in the basolateral compartment. Since EC SR-B1 deficiency does not affect the expression of genes that regulate inflammation in the aorta, or leukocyte-EC adhesion in basal or TNF-mediated pro-inflammatory conditions, a decrease in atherogenic lipoprotein profiles and atherosclerosis caused by EC SR-B1 expression suggests that EC SR-B1 might play a role in cholesterol efflux across ECs. SR-B1 expression is increased in atherosclerosis-prone regions of the mouse aorta before lesion formation, and in human atherosclerotic arteries when compared with normal arteries. In EC-intracellular vesicles, LDL particles colocalize with SR-B1, which interacting with dedicator of cytokinesis 4 (DOCK4), which is a receptor that recruits the guanine nucleotide exchange factor to its eight-amino-acid cytoplasmic domain. DOCK4 couples the SR-B1/LDL complex with activated Rac1, promoting the SR-B1/LDL internalization and thereby transporting LDL-cholesterol (LDL-C) into the arterial wall. Consequently, EC SR-B1 promotes atherosclerosis by facilitating LDL-C accumulation in the arterial wall [115••] (Fig. 3). Loss of EC PDZK1 (PDZK1^∆EC^) had no effect on lesion severity [115••], suggesting that EC SR-B1 mediates atherosclerosis via a PDZK1-independent mechanism without altering circulating lipids or vascular inflammation. Similarly, HDL action in CVD results in reduced activation of eNOS and an increase in ROS formation through low levels of PON1, an increase in MDA, and thus, oxidation of lipoproteins such as HDL and LDL [114] (Fig. 3). Ox-LDL can bind lectin-type oxidized LDL receptor 1 (LOX-1), resulting in promotion of atherosclerosis formation.

### Platelet SR-B1 Is Atheroprotective

In the atherosclerotic plaque development, the role of platelets is essential [116]. Activated platelets help to recruit immune cells, such as monocytes to injury sites on arterial walls. Macrophage stimulating factors expressed by platelets induce the monocytes and transformed them to activated macrophages. The activated macrophages induce apoptosis to the surrounding smooth muscle cells in the arterial wall. The arterial wall become weak and produce growth factors and free radicals which transform the normal, contractile smooth muscle cells to a proliferative stage [117]. As a result of these changes, those smooth muscle cells then migrate to the intimal layer of the arterial wall, proliferate, and narrow the lumen by creating plaques. The abnormal activation and aggregation of platelets is associated with increased atherosclerotic plaque formation and thus inhibiting that pathway can reduce risk of CVD [118]. Both the activated and resting platelets express SR-B1 and oxidized HDL binds to SR-B1 leading to inhibition of platelet activation and aggregation [119]. Additionally, natural HDL_3_ binds to SR-B1 on platelets and inhibits their activation [120].

## Potential Roles for SR-B1 And S1P in HDL-Independent Mechanisms of FC Efflux

Sphingosine 1-phosphate (S1P) plays a role in various biological functions including anti-apoptosis, anti-inflammation, pro-thrombosis, vasorelaxation, EC barrier functions, and the egression and activation of lymphocytes [121]. Low plasma S1P concentrations are associated with CVD. As a major carrier and acceptor, HDL binds and transports ~ 50–70% of plasma S1P. Plasma concentrations of HDL-S1P affect some HDL-mediated functions. Low HDL-S1P concentration is linked to atherosclerosis, CVD, MI, and renal insufficiency due to impaired HDL functions. Pharmacological increases in plasma HDL-S1P concentrations suppresses these adverse effects. However, the pathophysiological and molecular mechanisms by which changes in HDL-S1P concentrations are linked with these pathologies remain to be elucidated. Binding to HDL alters bioactivity and receptor presentation of S1P and thereby modulates S1P signaling. In the HDL-S1P complex, Apolipoprotein M (APOM) is a major S1P binding partner. The APOM-S1P complex modulates biological properties of S1P and metabolism of APOM-containing lipoproteins, which regulate plasma S1P concentration. S1P-APOM strengthens S1P anti-atherosclerotic properties while potentially weakening S1P pro-atherosclerotic properties, suggesting that APOM-S1P is an anti-atherogenic target.

S1P binding to its receptors (S1PRs) facilitate lipid transfer [122]. S1P/S1PRs regulate molecular pathways that control cellular homeostasis in multiple cell types including cardiac and vascular cells. Different cardiac S1PRs are implicated in different pathologies: S1PR1 is downregulated in experimental models of heart failure (HF), while its overexpression alleviates HF after MI; S1PR2 and 3 protect cardiomyocytes from ischemia/reperfusion damage. Dysregulated S1P/S1PRs is implicated in CVD, including atherosclerosis, coronary artery disease, MI, and HF. Similarly, SR-B1 also binds various S1PRs, required for the S1P-HDL-mediated pleiotropic effects of HDL [70, 123]. Using protein-fragment complementation assays and confocal microscopy approaches in primary rat aortic vascular smooth muscle and HEK293 cells, Lee et al. found that upon interaction with HDL, S1P triggers a transient SR-B1/S1PRs, thereby activating S1PRs and increasing intracellular calcium^25^. Both S1P and SR-B1 can bind S1PRs and HDL, and can be either anti- or pro-atherogenesis, suggesting that their paradoxical effects in atherogenesis could depend on their interacting partners. Although APOM is a major binding partner of S1P forming the HDL-S1P complex, other co-existing receptors can bind to S1P, particularly when plasma S1P concentration is acutely elevated. In such a context, biological properties modulated by S1P-signaling can be driven by HDL-independent mechanisms. One example is that the S1P-APOM association strengthens S1P-associated anti-atherosclerotic properties while potentially weakening S1P pro-atherosclerotic properties; whereas the S1P-Albumin association exerts both beneficial and detrimental effects on atherogenesis.

Adding to its well-accepted function as a cell surface HDL receptor that facilitates a selectively CE uptake [124–126], SR-B1 has also been implicated in various important biological functions including intracellular signaling, lipid accumulation, foam cell formation, and cellular apoptosis. For instance, SR-B1 modulates the susceptibility to LPS-induced tissue injury and plays a role in adaptive and innate immunity [127, 128]. SR-B1 recruits the non-receptor tyrosine kinase src to its C-terminal cytoplasmic tail, increasing src autophosphorylation leading to the phosphorylation and activation of AMP-activated protein kinase (AMPK). Although in other tissues, the PDZK1-SR-B1 association is required for SR-B1-HDL signaling, in brain microvascular ECs, PDZK1 binding is not required for HDL-C uptake. Internalized HDL partially co-localizes with SR-B1. However, in brain microvascular ECs, SR-B1 facilitates lipid transfer from and to HDL via a mechanism independent of HDL internalization and degradation [124–126]. Inhibition of eNOS promotes HDL internalization but raising NO concentrations does not affect HDL concentrations, suggesting that SR-B1-mediated HDL transcytosis in brain microvascular ECs is distinct from HDL-FC uptake [129]. This observation also supports SR-B1’s role beyond cholesterol efflux.

## FC Bioavailability Is Increased in SR-B1 KO Mice

Defective cholesterol transfer triggered by an increased FC bioavailability has been implicated in atherosclerosis. In mice, most lipoprotein-FC is rapidly cleared from the circulation (*t*_1/2_ < 5 min) without esterification. The remainder is taken up by various tissues and cells, especially erythrocytes. In SR-B1 KO vs. WT mice, diet-induced atherosclerosis is greater despite elevated plasma HDL concentrations. Similarly, a large population-based study showed that an increased plasma HDL concentration, in individuals who are heterozygous for the SR-B1 P376L mutation, is associated with increased CVD risks. In their study, Liu et al. noted a marked increase of HDL-FC mol% in plasma of the SR-B1 KO (41.1 mol%) as compared to that of the WT (16.0 mol%) mice [130••]. The SR-B1 KO show higher FC bioavailability for transfer to multiple tissues as compared to the WT mice. Plasma clearance of autologous HDL-FC mass is much slower in the SR-B1 KO as compared to the WT mice. Whereas FC efflux capacity is similar between the two genotypes, FC transfer from the HDL of SR-B1 KO vs. WT mice to LDL and to J774 macrophages was about 4-times greater. Moreover, in the SR-B1 KO, higher FC mol% in ovaries, erythrocytes, heart, and macrophages are associated with previously reported infertility in females, impaired cell maturation, cardiac dysfunction, and atherosclerosis. The FC contents of other tissues is similar between the SR-B1 KO and WT mice, and these tissues are not associated with any overt pathology. In addition, there were sex-dependent differences in tissue lipid composition and plasma FC clearance rates. This study highlights the importance of FC bioavailability in atherosclerosis and other pathologies [130••].

## Conclusion

Given the association of high plasma cholesterol concentrations to increased risk of CVD and atherosclerosis, past clinical studies have largely focused on increasing HDL-c content to induce RCT. As the primary receptor for HDL-c in the RCT pathway, SR-B1 is a crucial mediator of cholesterol transport. Although widely expressed in multiple tissues and cell types, SR-B1 expression and function is highly context-dependent [131]. Despite primarily being studied as an HDL-selective receptor, SR-B1 colocalizes with LDL particles in EC intracellular vesicles, allowing for active caveolae-mediated transcytosis of LDL. This observation challenges the long-held concept that LDL-C passively crosses permeable EC membranes and suggests that the inhibition of endothelial SR-B1-mediated delivery of LDL to arterial walls may be an effective therapy for CVD. Similarly, this study demonstrates the complexity surrounding SR-B1 context-dependent functionality; although it has long been thought to be primarily an atheroprotective mediator of RCT via HDL-c binding, SR-B1 can contribute negatively towards atherosclerosis progression in CVD through a multitude of pathways (Fig. 3).

This long-term focus on SR-B1-HDL-mediated cholesterol efflux through the hepatic RCT pathway underlies the current oversimplification of the physiological functions involving the SR-B1-HDL complex. Although current studies have largely focused on the role of HDL quantity in atheroprotection, the clinical failure of therapeutics that increase HDL-c concentration to have an effect upon CVD progression challenges the notion that HDL quantity is the primary mediator of atheroprotection [45–47]. Similarly, a recent study has demonstrated that a systemic SR-B1-KO in mice will dramatically increase HDL-c, but paradoxically raise CVD [130••]. These studies have revealed that plasma HDL-c concentration associates in a U-shaped relationship with CVD risk; while low HDL-c results in high risk of CVD related to hypertriglyceridemia [132, 133], those in the highest percentile for plasma HDL-c levels are also at risk for CVD. To explain this phenomenon, current studies should begin focusing on the quality and functionality of HDL-c rather than the quantity. Here, we review one proposal examining the FC bioavailability in HDL, resulting in a dysfunctional, atherogenic HDL-c that transfers FC back into macrophages, resulting in plaque formation. This hypothesis is supported by the study in SR-B1 KO mice and can be further examined clinically through the measurement of plasma HDL-c, and determination of HDL-FC and other phospholipid concentrations.

Other aspects of the SR-B1-HDL complex remain uncertain as well. Given the reduction in ABCG5/G8 expression in the SR-B1 KO mice, ABCG5/G8 may play a role in SR-B1-dependent biliary cholesterol secretion as well [130••]. While certain polymorphisms of ABCG5/G8 have been linked to reduction of HDL-C concentration [134], another meta-analysis examining common ABCG5/G8 polymorphisms has suggested that they demonstrate little association to markers of cholesterol homeostasis such as LDL-c levels [135]. Determination of a role for ABCG5/G8 in the SR-B1-HDL complex-dependent cholesterol secretion can further our understanding of this pathway. Similarly, given its reduction in SR-B1 KO mice, it is possible that low expression of ABCG5/G8 may contribute to FC bioavailability and the dysfunctionality of HDL-c.

Therefore, the FC bioavailability proposal firstly demonstrates the previous exaggerated focus on HDL-c’s involvement in RCT. Clinical validation of dysfunctional HDL with high FC bioavailability would lead to studies to elucidate the biological mechanisms underlying FC concentration for translational applications. Given the failures of therapies targeting HDL-c concentration in lowering CVD risk, future studies examining HDL-c functionality are likely to have more success in determining HDL-c importance in CVD risk.
